# Memory for Hand-Use Depends on Consistency of Handedness

**DOI:** 10.3389/fnhum.2013.00555

**Published:** 2013-09-04

**Authors:** James M. Edlin, Emily K. Carris, Keith B. Lyle

**Affiliations:** ^1^Department of Psychological and Brain Sciences, University of Louisville, Louisville, KY, USA

**Keywords:** handedness, interhemispheric interaction, self-performed tasks, enactment, episodic memory, action memory, individual differences

## Abstract

Individuals who do not consistently use the same hand to perform unimanual tasks (inconsistent-handed) outperform consistent right- and left-handed individuals on tests of episodic memory. We explored whether the inconsistent-hander (ICH) memory advantage extends to memory for unimanual hand use itself. Are ICHs better able to remember which hand they used to perform actions? Opposing predictions are possible, stemming from the finding that some regions of the corpus callosum are larger in ICHs, especially those that connect motor areas. One hypothesis is that greater callosally mediated interhemispheric interaction produces ICHs’ superior retrieval of episodic memories, and this may extend to episodic memories for hand use. Alternatively, we also hypothesized that greater interhemispheric interaction could produce more bilateral activation in motor areas during the performance and retrieval of unimanual actions. This could interfere with ICHs’ ability to remember which hand they used. To test these competing predictions in the current study, consistent- and inconsistent-handers performed unimanual actions, half of which required manipulating objects and half of which did not. Each action was performed four times in one of five conditions that differed in the ratio of left to right hand use: always left (4:0), usually left (3:1), equal (2:2), usually right (1:3), or always right (0:4). We compared consistent- and inconsistent-handers on recall of the left:right ratio for each action. ICHs remembered how they performed actions better than consistent-handers, regardless of ratio. These findings provide another example of superior episodic retrieval in ICHs. We discuss how greater interaction might benefit memory for hand use.

## Introduction

Individuals differ in the consistency with which they use a single preferred hand to perform unimanual tasks. Some individuals are highly consistent while others are relatively inconsistent, making greater use of both hands. We refer to this interindividual variable as handedness consistency, although it might also be called manual lateralization. Of interest to memory researchers, degree of handedness consistency, as measured by self-report, predicts performance on tests of episodic memory (for review, see Prichard et al., 2013). On average, inconsistent individuals remember events more accurately and with greater subjective vividness and detail than do consistent individuals.

Why is handedness consistency related to memory? The dominant theory in the literature is based on two assumptions: (1) inconsistent-handers (ICHs) have greater interhemispheric interaction than consistent-handers (CHs), and (2) greater interhemispheric interaction enhances some types of memory retrieval (Christman and Propper, 2001). The first assumption is tenuously supported by consistency-based anatomical differences in the corpus callosum. The corpus callosum is the major pathway for communication between the left and right cerebral hemispheres and has sometimes been found to be larger in ICHs than CHs (Witelson, 1985; Habib et al., 1991; Cowell et al., 1993; Luders et al., 2010), but not always (Jäncke and Steinmetz, 2003; Welcome et al., 2009). Differences in measurement techniques and measurement of different subregions of the corpus callosum may have led to these discrepancies. Because the corpus callosum is a bundle of fibers that branch to different cortical regions, consistency-based differences may exist in some callosal regions, but not others (Nowicka and Tacikowski, 2011). For example, Luders et al., found differences only in the anterior and posterior midbody of the corpus callosum, which primarily connect the sensory-motor cortices (Hofer and Frahm, 2006).

Behavioral studies provide more definitive evidence that ICHs have greater interhemispheric interaction than CHs. Interhemispheric transfer time, measured as the difference in response times for information processed in the hemisphere opposite the response hand and information processed in the same hemisphere as the response hand, is shorter in ICHs than CHs (Cherbuin and Brinkman, 2006; Bernard et al., 2011). Furthermore, ICHs exhibit greater interhemispheric transfer of skill learning (Chase and Seidler, 2008). Finally, Lyle and Martin (2010) found that ICHs were more accurate than CHs at detecting matches between letters (e.g., *A* and *a*) that were briefly flashed to separate visual fields.

The second assumption of the interhemispheric interaction hypothesis has sometimes (see Christman and Propper, 2001) been grounded in the hemispheric encoding/retrieval asymmetry (HERA) model (Tulving et al., 1994), according to which episodic memory is left lateralized in frontal regions at encoding and right lateralized at retrieval. If this is the case, then increased interhemispheric interaction could improve episodic retrieval by enhancing the transfer of information from the left to the right hemisphere. It appears, however, that the HERA model may only hold for relatively simple episodic retrieval tasks that require primarily familiarity-based judgments. Extensive neuroimaging evidence shows that more complex tasks that require recall of specific episodic details produce frontal activation bilaterally during retrieval (see Nolde et al., 1998; Miller et al., 2002). From this perspective, greater interhemispheric interaction may improve retrieval by enhancing coordination of retrieval areas across the hemispheres (Lyle et al., 2008b), but this should apply only to more complex tasks. Findings have so far supported this prediction with consistency-based differences limited to tasks that require recall. These tasks have included free recall (e.g., Propper et al., 2005; Lyle et al., 2008a; Christman and Butler, 2011), cued recall (Parker and Dagnall, 2010), associative recognition (Lyle et al., 2008b), and recall of source information (Christman et al., 2004; Lyle et al., 2008b). Differences have not been found on tasks with a small or non-existent recall component, including old/new recognition (Propper and Christman, 2004; Lyle et al., 2008a), short-term digit span (Lyle et al., 2008b), and implicit word-fragment completion (Propper et al., 2005).

Despite abundant evidence for consistency-based differences in episodic memory, memory for hand use itself has never been examined. If individuals perform unimanual actions multiple times with different combinations of their left and right hands, does their ability to recall how often they used a particular hand for each task depend on their consistency in everyday life? The question is of interest for three reasons. First, it is currently unclear to what extent consistency-based differences in memory occur for non-verbal information, including motoric and frequency information. Most studies that have documented a memory advantage for ICHs have employed word stimuli. We count among these Lyle et al.’s (2008b) tests of source memory. Although the tests revealed that ICHs were more likely than CHs to remember non-verbal source information, subjects were remembering the source of words. The only example of an inconsistently handed memory advantage for strictly non-verbal stimuli is Lyle and Jacobs’s (2010) finding that ICHs were less likely than CHs to falsely remember visual details from a slideshow depicting a complex event. Although suggestive, an inconsistently handed advantage occurred in only one of two studies Lyle and Jacobs conducted. The studies had somewhat different procedures and Lyle and Jacobs reasoned that the advantage occurred in the procedure that necessitated greater recall of episodic details. Nonetheless, we consider this single result only preliminary evidence for an inconsistently handed memory advantage for non-verbal stimuli. Note also that, when Lyle et al., tested ICHs and CHs on memory for faces in an old/new recognition procedure, they did not find an advantage for ICHs. The authors attributed this null effect to the fact that interhemispheric interaction plays little role in face recognition (Gazzaniga and Smylie, 1983), but we cannot rule out that it was due to the non-verbal nature of the stimuli. If ICHs’ memory advantage does extend to non-verbal information, then we would expect it to occur on a test of memory for the ratio of left to right-hand usage because such a test clearly requires *specific* recall.

The second reason for our interest in memory for hand use is that the hemispheric basis of unimanual action has been found to differ between ICHs and CHs and, from this, one could predict an inconsistently handed memory *dis*advantage. When using the dominant hand, both CHs and ICHs activate the contralateral motor cortex, but ICHs show greater activation of the ipsilateral motor cortex than CHs (Dassonville et al., 1997; Bernard et al., 2011). Greater ipsilateral activation may be related to the increased thickness of the corpus callosum that connects motor regions in ICHs. Callosal connections appear to be involved in spreading activation from one hemisphere to the other (e.g., Kinsbourne, 2003; Bloom and Hynd, 2005). When ICHs execute unimanual actions, greater callosal connectivity may cause activity in the controlling motor cortex (contralateral to the hand in use) to spread to the ipsilateral cortex. It is possible that this would have negative consequences for memory for hand use. Retrieval of action memories is associated with reactivation of primary motor areas that were active while performing the actions (Nyberg et al., 2001). This reactivation during retrieval is thought to aid memory for actions themselves (Masumoto et al., 2006), producing superior memory for self-performed actions than for verbally encoded action phrases (for review, see Cohen, 1989) or observed actions (e.g., Hornstein and Mulligan, 2001). Presumably, reactivation may also help individuals remember which hand they used to perform actions, with activity in a given hemisphere providing evidence that the action was performed with the contralateral hand. However, CHs and ICHs may not benefit equally from reactivation. Conceivably, ICHs’ greater bilateral activation during unimanual action could be mirrored in greater bilateral activation during retrieval, causing confusion about which hand was used. More strictly unilateral activation/reactivation among CHs could lead to greater precision in memory.

The third and final reason for our interest in memory for hand use is that the study of consistency-based differences in memory has rested on the critical assumption that people can accurately remember how they use their hands in everyday life. In this area of research, consistency has invariably been measured by self-report on hand preference inventories. On these inventories, subjects report the frequency with which they use one hand or the other to perform everyday unimanual tasks (e.g., brushing one’s teeth). These are reports of past behavior and therefore constitute a type of memory judgment. These judgments are generally assumed to be accurate. In other words, individuals who report consistency (or inconsistency) are assumed to actually perform everyday tasks in a consistent (or inconsistent) manner. However, memory is fallible in many respects and remembering contextual information, as opposed to item information, can be especially challenging (Johnson et al., 1993). It may be that people can remember that they performed certain actions (item information) without remembering how they performed them (contextual information). Therefore, we sought to conduct an initial investigation of memory for hand use. Although hand preference inventories probe memory for actions performed outside the laboratory, we felt a reasonable first step was to test memory for actions performed in a controlled laboratory setting.

In sum, our goal was to determine the relationship between handedness consistency and memory for hand use. ICHs’ putatively greater interhemispheric interaction could give them an advantage over CHs if it facilitates recall of episodic detail. Alternatively, greater interaction between motor areas, and possible bilateral reactivation of these regions during retrieval, could reduce ICHs’ ability to determine which hand or hands they used^1^. Given the centrality of handedness inventories in this research area, we modeled our memory test after the structure of those inventories. Namely, we measured memory for left:right hand-use ratios for actions. In addition, we examined memory for actions performed with and without objects. Hand preference inventories primarily consist of questions about actions performed with objects, so these types of actions were of greatest interest to us. However, we were also interested for exploratory purposes in whether the presence of an object would affect memory for hand-use or moderate any consistency-based memory differences.

## Materials and Methods

### Subjects

Subjects were undergraduates aged 18–30 who received credit in psychology courses for participating and provided informed consent under protocols approved by the University of Louisville IRB. Using the handedness inventory described below, and following the method from previous studies (e.g., Lyle et al., 2008b, 2012; Edlin and Lyle, 2013), we classified subjects according to their inventory scores. Subjects were classified as CHs if the absolute value of their inventory score was 80 or greater (*n* = 50, *M* absolute score = 92.8, eight males) or as ICHs if the absolute value of their inventory score was<80 (*n* = 35, *M* absolute score = 57.6, nine males, one unknown). Although we categorized subjects by consistency instead of direction of handedness, our sample included seven subjects who could be classified as left-handed due to negative inventory scores. Of these, two were CHs (*M* score = −92.5, one male) and five were ICHs (*M* score = −59, two males, one unknown).

### Materials

We assessed degree of handedness consistency using a modified version of Oldfield’s (1971) Edinburgh Handedness Inventory. The inventory queries hand preferences for 10 activities (writing, drawing, using a spoon, opening jars, using a toothbrush, throwing, combing hair, using scissors, using a knife without a fork, and striking a match). For each activity, the response options (and corresponding point values) are Always Right (+10), Usually Right (+5), No Preference (0), Usually Left (−5), and Always Left (−10). Inventory scores range from −100 (consistently left-handed) to +100 (consistently right-handed) in 5-point increments.

For the hand-use task, we selected 20 actions (see Table 1), half of which required manipulating objects (e.g., roll the dice) and half of which did not (e.g., snap your fingers). The necessary objects were provided to subjects in a container at the beginning of the experiment. Actions were performed at one of five possible left:right hand-use ratios: always left (4:0), usually left (3:1), equal (2:2), usually right (1:3), or always right (0:4). Assignment of action to ratio was counterbalanced. For the performance/encoding phase of the procedure, we created four blocks of action commands. Each command instructed subjects to perform 1 of the 20 actions with a particular hand (e.g., “roll the dice with your left hand”). Thus, there were 20 commands per block. The commands were presented in pseudo-random order such that object actions and no-object actions were evenly distributed throughout each block. Commands were presented in a different order in each block. For actions assigned to the always-left and always-right ratios, the command was to use the same hand in every block. For actions assigned to the other three ratios, the commands varied in accordance with the particular ratio. Among actions assigned to the same ratio, the sequence of left/right commands was different for each action. For the retrieval phase of the procedure, a new random ordering of the 20 actions (without a performance command) was created for each subject.

**Table 1:** **Actions performed during the hand-use task**.

Without objects	With objects^a^
Blow a kiss	Bounce the ball (small rubber ball)
Count to five	Drop a coin in the box (small box with slot and four pennies)
Cover your eye	Flip over the card (playing card)
Give a thumbs up	Move the mouse in a circle (mouse with cord removed)
Knock on the desk	Open the tupperware (small rubbermaid container)
Pat your head	Pull out your chair
Point to the monitor	Roll the die (six-sided die)
Snap your fingers	Take a piece of tape (roll of scotch tape)
Squeeze your hand	Take off the marker cap (dry erase marker)
Wave	Use the hole puncher (single hole punch and index card)

*^a^Subjects received the objects included in parenthesis*.

### Procedure

Subjects first completed the handedness inventory and were given the container of objects. Subjects then began the performance/encoding phase. Action commands appeared on a computer screen one at a time. For actions that required an object, subjects took the object out of the container, performed the action with the specified hand a single time, and replaced the object in the container. For no-object actions, subjects simply performed the action one time. Subjects were instructed to perform each action four times. Left:right hand-use ratio varied between actions such that each subject performed some actions in all five of the ratios. After each action, subjects rated the action on how difficult it was to perform the action with the specified hand and how natural it felt to do so. Ratings were made on a Likert scale ranging from 1 (*difficult*/*unnatural*) to 9 (*easy*/*natural*). Performance and ratings were self-paced. Subjects performed all 20 actions in each of 4 blocks. After the fourth and final block, there was a surprise memory test. Each action was presented on the screen and subjects were instructed to select the hand-use ratio they remembered using to perform the task by pressing a number one through five that corresponded to always left, usually left, equal, usually right, or always right. Subjects were required to choose a ratio for each action.

## Results

### Recall

We submitted proportion of correct responses on the hand-use memory test to a 2 (consistency: CH or ICH) × 5 (ratio: always left, usually left, equal, usually right, or always right) × 2 (action type: object or no object) mixed-factorial ANOVA with consistency as a between-subjects factor and ratio and action type as within-subjects factors.

**Figure 1:**
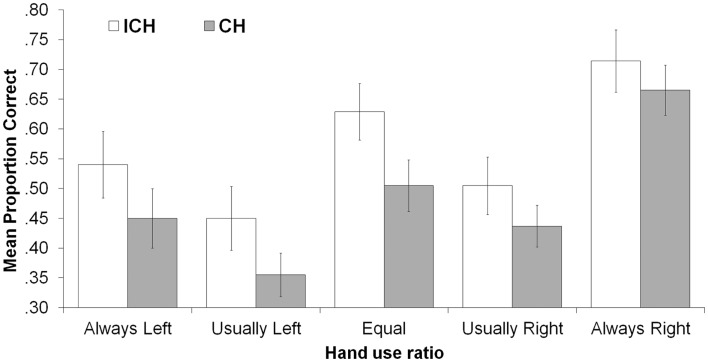
**Mean proportion correct as a function of hand-use ratio and consistency**. Error bars indicate ±1 SEM.

Addressing our primary research question, there was a main effect of consistency with ICHs (*M* = 0.57) producing more correct responses than CHs (*M* = 0.47), *F*(1, 83) = 4.38, *p* = 0.039, $\eta_{p}^{2}=0.05$. ICHs remembered all ratios numerically better than did CHs, as shown in Figure 1 and as reflected in the non-significant consistency X ratio interaction, *F*(4, 80) = 0.232, *p* = 0.920, $\eta_{p}^{2}=0.011$. The consistency X action type interaction was also non-significant, *F*(1, 83) = 1.41, *p* = 0.239, $\eta_{p}^{2}=0.017$, indicating that the inconsistently handed advantage occurred regardless of whether actions were performed with or without objects.

**Figure 2:**
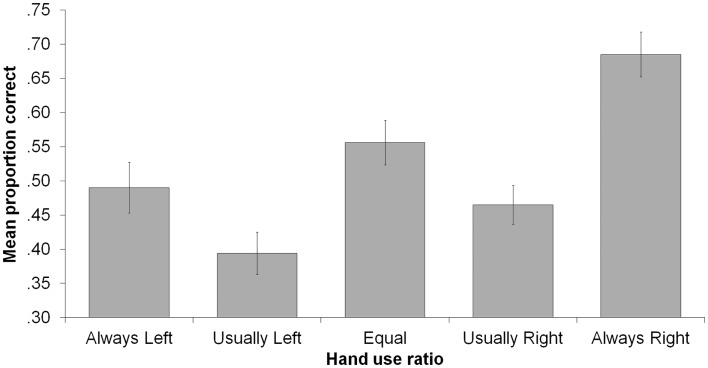
**Mean proportion correct as a function of hand-use ratio**. Error bars indicate ±1 SEM.

**Figure 3:**
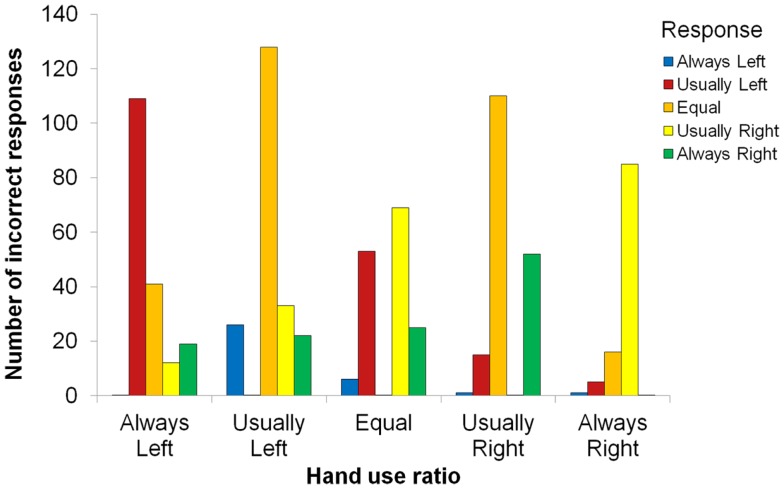
**Distribution of incorrect responses as a function of hand-use ratio**.

There was also a main effect of ratio, *F*(4, 80) = 12.72, *p* < 0.001, $\eta_{p}^{2}=0.389$, indicating that some hand-use behaviors were better remembered than others (see Figure 2). Most strikingly, proportion correct for the always-right ratio (*M* = 0.68) was significantly higher than for all other ratios, smallest *t*(84) = 2.97, *p* = 0.004. Also, proportion correct was higher for the equal ratio (*M* = 0.56) than for the usually left (*M* = 0.39) or usually right (*M* = 0.47) ratios, smallest *t*(84) = 2.34, *p* = 0.022. In addition, proportion correct for usually right was higher than for usually left, *t*(84) = 2.16, *p* = 0.033, and always left (*M* = 0.49) was higher than usually left, *t*(84) = 2.20, *p* = 0.030. We examined the distribution of incorrect responses, which is shown in Figure 3. Three patterns are evident. First, when the correct ratio was unequal, incorrect responses were usually shifted to a less extreme (versus more extreme) ratio than the actual one. Second, when the correct ratio was equal, incorrect responses tended to be shifted to the usually left or usually right ratio (rather than to one of the always ratios). Third, there was a slight tendency to respond with ratios in which there was more right-hand usage (rather than with ratios in which there was more left-hand usage); this can be seen most clearly in the equal ratio condition. None of these patterns suggest a strong response bias that could account for any of the significant between-ratio accuracy differences.

The ratio X action type interaction approached significance, *F*(4, 80) = 2.32, *p* = 0.058, $\eta_{p}^{2}=0.027$, but the trend was not readily interpretable. Briefly, proportion correct with and without objects was very similar and statistically indistinguishable for all ratios except usually right, for which object actions (*M* = 0.55) were remembered better than no-object actions (*M* = 0.40), *t*(84) = 2.63, *p* = 0.01.

### Ratings

Because difficulty and naturalness ratings were highly correlated, *r*(85) = 0.53, *p* < 0.001, we combined the two ratings into a composite fluency rating. We submitted ratings to a 2 (hand: left or right) × 2 (action type: object or no object) × 2 (consistency: CH or ICH) mixed-factorial ANOVA with hand and action type as within-subjects factors and consistency as a between-subjects factor.

The main effect of consistency was not significant, *F*(1, 83) = 1.08, *p* = 0.302, $\eta_{p}^{2}$ = 0.013, and neither were the interactions between consistency and hand or action type, *F*s < 1, suggesting that the inconsistently handed memory advantage described above was not due to greater fluency when performing the actions.

There were significant main effects of hand, *F*(1, 83) = 121.24, *p* < 0.001, $\eta_{p}^{2}$ = 0.594, and action type, *F*(1, 83) = 129.028, *p* < 0.001, $\eta_{p}^{2}$ = 0.609, but these were qualified by a significant interaction between the two factors, *F*(1, 83) = 68.479, *p* < 0.001, $\eta^{2}$ = 0.452. Overall, subjects felt more fluent when performing right-hand actions (*M* = 8.7) than left-hand actions (*M* = 7.5), and when performing actions without objects (8.4) than with them (7.9). However, for right-hand actions, the fluency difference between actions performed without an object (*M* = 8.8) versus with one (*M* = 8.7) was very small, albeit significant, *t*(84) = 4.10, *p* < 0.001. For left-hand actions, the difference between actions performed without an object (*M* = 7.9) versus with one (*M* = 7.2) was markedly larger, *t*(84) = 10.85, *p* < 0.001.

## Discussion

The primary goal of this study was to compare the ability of ICHs and CHs to remember how they used their hands. Recent studies have found that ICHs have superior episodic memory but have not revealed whether this advantage extends to non-verbal stimuli or to memory for hand use, in particular. Empirically, many studies have shown an inconsistently handed advantage for verbal stimuli (e.g., Propper et al., 2005; Lyle et al., 2008a; Christman and Butler, 2011), but, to our knowledge, only one has done the same for non-verbal stimuli (Lyle and Jacobs, 2010). Given that ICHs differ from CHs in language lateralization (Knecht et al., 2000) and that ICHs have more diffuse semantic networks (Sontam and Christman, 2012), it was conceivable that these factors resulted in a memory advantage specifically for verbal stimuli. Theoretically, ICHs’ putatively greater interhemispheric interaction (Christman and Propper, 2001; Lyle et al., 2008b) fostered opposing predictions about memory for hand use. Greater interaction could benefit memory by increasing recall of episodic details or harm it by producing bilateral reactivation in motor cortex during retrieval of unimanual actions. Our results resolved these empirical and theoretical uncertainties by clearly showing that ICHs performed better than CHs on our test of memory for hand-use ratio. This finding supports the conclusion that the inconsistently handed memory advantage does extend to non-verbal events, including self-performed actions.

Although we found a consistency-based difference in memory for hand use, elucidating the cause of this difference requires additional research. The results of our ratings data argue against the possibility that the memory difference was due to differences between ICHs and CHs in hand-use fluency, because the groups provided similar fluency ratings. Following, we consider three other possible explanations for ICHs’ superior ability to remember which hand they used to perform actions. One is that, while reactivation of motor cortex at retrieval can potentially be used by either CHs or ICHs to determine which hand or hands were used at performance/encoding, ICHs are more skilled at interpreting this reactivation. If ICHs routinely experience bilateral activation during performance (Dassonville et al., 1997) and corresponding bilateral reactivation during retrieval (Nyberg et al., 2001), they may have adapted some means of differentiating between contralateral activation due to hand-use and ipsilateral activation from callosal “overflow.” This could make ICHs more sophisticated decision makers than CHs when recalling which hand or hands they used to perform an action during instances of bilateral motor cortex reactivation.

Alternatively, individuals may use motor cortex reactivation only to determine that an action was performed versus not, and not to determine exactly which hand or hands were used to perform the action. Prior studies showing a memory advantage for performed actions compared to actions encoded using other methods (e.g., reading action phrases; see Cohen, 1989) have focused on remembering the presence or absence of an action and not specific details of the action. If specific hand-use information is not gleaned from motor cortex reactivation, then correctly remembering hand use would presumably rely on recollecting specific episodic details encoded during the act of performance (e.g., visual details, cognitive operations). If this explanation is correct, we could attribute the superior performance of ICHs on this task to the already established fact that ICHs are superior to CHs when recalling episodic details.

A third possible explanation for ICHs’ superior memory for hand-use assumes, like our first explanation, that motor cortex reactivation at retrieval can serve as a useful source of information about which hand or hands were used, but that ICHs are less reliant on it than CHs. ICHs’ hand-use memories may be based on both motor cortex reactivation and, as proposed in our second explanation, recall of additional episodic details. In contrast, CHs may rely largely on motor cortex reactivation, which may be insufficient to remember specific hand-use ratios. Propper and Christman (2004) found that ICHs’ memories are more likely than CHs’ to be accompanied by a rich recollective experience (indexed by “Remember” responses), whereas CHs’ memories are more likely than ICHs’ to be accompanied only by a non-specific sense of familiarity (indexed by “Know” responses). In the context of remembering hand use, CHs’ reliance on motor cortex reactivation may give rise to a similar sense of familiarity while ICHs’ use of additional episodic details may produce a sense of recollection.

Consideration of these possibilities raises the important lingering question of exactly how ICHs’ putatively greater interhemispheric interaction might cause them to remember the episodic past more accurately and in greater detail. One idea not yet put forth in the literature is that ICHs’ threshold for recruiting both hemispheres, versus only one, during episodic memory tests may be lower than CHs’. As mentioned in the introduction, complex memory tasks produce bilateral frontal activation (see Nolde et al., 1998; Miller et al., 2002) and greater connectivity between the hemispheres via the corpus callosum has been proposed as an explanation for ICHs’ superior memory performance on these tasks. However, recruitment of both hemispheres to perform a task comes at a cost, which is dependent on interhemispheric transfer times. Banich (1998) proposed that simple tasks with low attentional demands are more efficiently processed by a single hemisphere specialized for that task. As task complexity increases, processing load overcomes the cost of transferring information across the corpus callosum. The threshold at which it is more efficient to recruit both hemispheres than rely on a single specialized hemisphere would theoretically be lower for individuals who have faster interhemispheric transfer times, including ICHs (Cherbuin and Brinkman, 2006; Bernard et al., 2011). Therefore, in addition to ICHs having an advantage on memory tasks that typically induce bilateral processing in all individuals (ICHs and CHs alike), ICHs may also be more likely to recruit additional hemispheric processing capability on tasks are not typically associated with bilateral activation (that is, not associated with bilateral activation in CHs, who constitute the majority of the population).

Our finding that ICHs were better at recalling hand-use ratios than CHs might be considered worrisome given that, in research examining the relationship between consistency and cognition (as well as consistency and personality; e.g., Christman et al., 2008; Lyle and Grillo, 2013) classification as ICHs or CHs is based on self-reported hand use. If ICHs are superior to CHs at recalling instances in which they used a combination of their left and right hands to perform tasks in everyday life, as they were better at remembering these instances in the laboratory, then a troubling possibility presents itself. ICHs and CHs could conceivably have similar real-world hand-use behaviors for the actions queried on hand preference inventories, including a similar rate of inconsistent behavior, but ICHs may be more likely to remember that they behaved inconsistently. If this were the case, it would undercut the idea that people who use their hands inconsistently have better episodic memory, and instead mean that people who have better episodic memory are more likely to remember instances of inconsistency when completing handedness questionnaires. Of course, recalling the exact left:right ratios for laboratory tasks performed only four times and after a retention interval of only a few minutes is different in many respects than recalling one’s pattern of behavior for real-world actions performed innumerable times outside of the laboratory. Nonetheless, ICHs have been found to have more accurate and more detailed memories for events outside of the lab (Christman et al., 2003; Propper et al., 2005; Parker and Dagnall, 2010). Of course, it is also possible that people rely more on semantic memory instead of retrieving specific events when recalling patterns of behavior for real-world actions. There is some evidence that consistency-based differences do not extend to semantic memory (Propper et al., 2005), therefore ICHs and CHs may be equally accurate when reporting hand-use on handedness inventories. Future research is needed to explore the accuracy of responses on handedness questionnaires.

Finally, we consider our finding that, regardless of consistency, some hand-use ratios were remembered better than others. In particular, the always-right ratio was remembered significantly better than all others. In interpreting this finding, recall that our sample included only seven left-handed subjects (i.e., subjects with negative scores on the handedness inventory). The strong representation of right-handers was evident in the subjective fluency data where subjects rated left-hand actions as being more difficult and less natural than right-hand actions. Hence, most subjects in our sample, regardless of consistency, were right-hand dominant and we found that memory was best for actions that were always performed with the dominant hand. This may be due to the strength of motor cortex reactivation during recall. Prior studies have reported greater activation in the contralateral hemisphere when performing tasks with the dominant hand than with the non-dominant hand (Dassonville et al., 1997). If this pattern is mirrored in reactivation at retrieval, and individuals rely on reactivation to determine the hand used to perform an action, then stronger reactivation following right- than left-hand actions should have resulted in superior memory for always-right actions than always-left actions. This is the result we obtained. For actions performed with both hands, both motor cortices may have been reactivated, but there may have been stronger reactivation in the dominant (left) hemisphere than in the non-dominant (right) hemisphere, and the former may have masked the latter. This could lead to failures to remember that these actions were performed with both hands, reducing accuracy for the usually left, equal, and usually right ratios.

Also, right-handers have been found to exhibit bilateral activation in motor cortex when performing tasks with their left hand (e.g., Kim et al., 1993; Cramer et al., 1999). The unexpected ipsilateral (left hemisphere) activation may be related to the increased complexity of using the non-dominant hand (Haaland et al., 2004). If bilateral activation during performance is mirrored at retrieval, and individuals use patterns of reactivation to determine which hand was used, this could have contributed to poorer memory for always-left actions than always-right actions.

In summary, we compared the ability of ICHs and CHs to remember left:right hand-use ratios for actions and found that ICHs outperformed CHs. Both groups were significantly better at remembering the actions performed in the always-right ratio than all other ratios.

## Conflict of Interest Statement

The authors declare that the research was conducted in the absence of any commercial or financial relationships that could be construed as a potential conflict of interest.

## Footnotes

^1^ These two possibilities are of primary interest to the authors, but other predictions are certainly possible. For example, one might have expected CHs to have superior memory relative to ICHs for actions performed with the left hand due to the greater distinctiveness of left-hand usage for the former group than the latter (remembering that the vast majority of our subjects were right-hand dominant).

## References

[B1] Banich M. T. (1998). The missing link: the role of interhemispheric interaction in attentional processing. Brain Cogn. 36, 128–157 10.1006/brcg.1997.0950 9520311

[B2] Bernard J. A. Taylor S. F. Seidler R. D. (2011). Handedness, dexterity, and motor cortical representations. J. Neurophysiol. 105, 88–99 10.1152/jn.00512.2010 20943944

[B3] Bloom J. S. Hynd G. W. (2005). The role of the corpus callosum in interhemispheric transfer of information: excitation or inhibition? Neuropsychol. Rev. 15, 59–71 10.1007/s11065-005-6252-y 16211466

[B4] Chase C. Seidler R. (2008). Degree of handedness affects intermanual transfer of skill learning. Exp. Brain Res. 190, 317–328 10.1007/s00221-008-1472-z 18592225 PMC2570758

[B5] Cherbuin N. Brinkman C. (2006). Hemispheric interactions are different in left-handed individuals. Neuropsychology 20, 700–707 10.1037/0894-4105.20.6.700 17100514

[B6] Christman S. D. Butler M. (2011). Mixed-handedness advantages in episodic memory obtained under conditions of intentional learning extend to incidental learning. Brain Cogn. 77, 17–22 10.1016/j.bandc.2011.07.003 21807450

[B7] Christman S. D. Garvey K. J. Propper R. E. Phaneuf K. A. (2003). Bilateral eye movements enhance the retrieval of episodic memories. Neuropsychology 17, 221–229 10.1037/0894-4105.17.2.221 12803427

[B8] Christman S. D. Henning B. Geers A. L. Propper R. E. Niebauer C. L. (2008). Mixed-handed persons are more easily persuaded and are more gullible: interhemispheric interaction and belief updating. Laterality 13, 403–426 10.1080/13576500802079646 18608851

[B9] Christman S. D. Propper R. E. (2001). Superior episodic memory is associated with interhemispheric processing. Neuropsychology 15, 607–616 10.1037/0894-4105.15.4.607 11761050

[B10] Christman S. D. Propper R. E. Dion A. (2004). Increased interhemispheric interaction is associated with decreased false memories in a verbal converging semantic associates paradigm. Brain Cogn. 56, 313–319 10.1016/j.bandc.2004.08.005 15522769

[B11] Cohen R. L. (1989). Memory for action events: the power of enactment. Educ. Psychol. Rev. 1, 57–80 10.1007/BF01326550

[B12] Cowell P. E. Kertesz A. Denenberg V. H. (1993). Multiple dimensions of handedness and the human corpus callosum. Neurology 43, 2353–2357 10.1212/WNL.43.11.2353 8232955

[B13] Cramer S. C. Finklestein S. P. Schaechter J. D. Bush G. Rosen B. R. (1999). Activation of distinct motor cortex regions during ipsilateral and contralateral finger movements. J. Neurophysiol. 81, 383–387 9914297 10.1152/jn.1999.81.1.383

[B14] Dassonville P. Zhu X. H. Ugurbil K. Kim S. G. Ashe J. (1997). Functional activation in motor cortex reflects the direction and the degree of handedness. Proc. Natl. Acad. Sci. U.S.A. 94, 14015–14018 10.1073/pnas.94.25.14015 9391144 PMC28424

[B15] Edlin J. M. Lyle K. B. (2013). The effect of repetitive saccade execution on the attention network test: enhancing executive function with a flick of the eyes. Brain Cogn. 81, 345–351 10.1016/j.bandc.2012.12.006 23485024

[B16] Gazzaniga M. S. Smylie C. S. (1983). Facial recognition and brain asymmetries – clues to underlying mechanisms. Ann. Neurol. 13, 536–540 10.1002/ana.410130511 6870204

[B17] Haaland K. Y. Elsinger C. L. Mayer A. R. Durgerian S. Rao S. M. (2004). Motor sequence complexity and performing hand produce differential patterns of hemispheric lateralization. J. Cogn. Neurosci. 16, 621–636 10.1162/089892904323057344 15165352

[B18] Habib M. Gayraud D. Oliva A. Regis J. Salamon G. Khalil R. (1991). Effects of handedness and sex on the morphology of the corpus callosum – a study with brain magnetic resonance imaging. Brain Cogn. 16, 41–61 10.1016/0278-2626(91)90084-L 1854469

[B19] Hofer S. Frahm J. (2006). Topography of the human corpus callosum revisited – comprehensive fiber tractography using diffusion tensor magnetic resonance imaging. Neuroimage 32, 989–994 10.1016/j.neuroimage.2006.05.044 16854598

[B20] Hornstein S. L. Mulligan N. W. (2001). Memory of action events: the role of objects in memory of self- and other-performed tasks. Am. J. Psychol. 114, 199–217 10.2307/1423515 11430149

[B21] Jäncke L. Steinmetz H. (2003). “Brain size: a possible source of interindividual variability in corpus callosum morphology,” in The Parallel Brain: The Cognitive Neuroscience of the Corpus Callosum, eds Zaidel E. Iacoboni M. (Cambridge, MA: MIT Press), 51–63

[B22] Johnson M. K. Hashtroudi S. Lindsay D. S. (1993). Source monitoring. Psychol. Bull. 114, 3–28 10.1037/0033-2909.114.1.3 8346328

[B23] Kim S. G. Ashe J. Hendrich K. Ellermann J. M. Merkle H. Ugurbil K. (1993). Functional magnetic resonance imaging of motor cortex – hemispheric asymmetry and handedness. Science 261, 615–617 10.1126/science.8342027 8342027

[B24] Kinsbourne M. (2003). “The corpus callosum equilibrates the cerebral hemispheres,” in The Parallel Brain: The Cognitive Neuroscience of the Corpus Callosum, eds Zaidel E. Iacoboni M. (Cambridge, MA: MIT Press), 271–281

[B25] Knecht S. Drager B. Deppe M. Bobe L. Lohmann H. Floel A. (2000). Handedness and hemispheric language dominance in healthy humans. Brain 123, 2512–2518 10.1093/brain/123.12.2512 11099452

[B26] Luders E. Cherbuin N. Thompson P. M. Gutman B. Anstey K. J. Sachdev P. (2010). When more is less: associations between corpus callosum size and handedness lateralization. Neuroimage 52, 43–49 10.1016/j.neuroimage.2010.04.016 20394828 PMC2903194

[B27] Lyle K. B. Grillo M. C. (2013). Consistent-handed individuals are more authoritarian. Laterality. Available at: http://pubget.com/paper/23586369 10.1080/1357650X.2013.783044 23586369

[B28] Lyle K. B. Hanaver-Torrez S. D. Häcklander R. P. Edlin J. M. (2012). Consistency of handedness, regardless of direction, predicts baseline memory accuracy and potential for memory enhancement. J. Exp. Psychol. Learn. Mem. Cogn. 38, 187–193 21843021 10.1037/a0024831

[B29] Lyle K. B. Jacobs N. E. (2010). Is saccade-induced retrieval enhancement a potential means of improving eyewitness evidence? Memory 18, 581–594 10.1080/09658211.2010.493891 20658433

[B30] Lyle K. B. Logan J. M. Roediger H. L. (2008a). Eye movements enhance memory for individuals who are strongly right-handed and harm it for individuals who are not. Psychon. Bull. Rev. 15, 515–520 10.3758/PBR.15.3.515 18567248

[B31] Lyle K. B. McCabe D. P. Roediger H. L. (2008b). Handedness is related to memory via hemispheric interaction: evidence from paired associate recall and source memory tasks. Neuropsychology 22, 523–530 10.1037/0894-4105.22.4.523 18590363

[B32] Lyle K. B. Martin J. M. (2010). Bilateral saccades increase intrahemispheric processing but not interhemispheric interaction: implications for saccade-induced retrieval enhancement. Brain Cogn. 73, 128–134 10.1016/j.bandc.2010.04.004 20452714

[B33] Masumoto K. Yamaguchi M. Sutani K. Tsuneto S. Fujita A. Tonoike M. (2006). Reactivation of physical motor information in the memory of action events. Brain Res. 1101, 102–109 10.1016/j.brainres.2006.05.033 16782071

[B34] Miller M. B. Kingstone A. Gazzaniga M. S. (2002). Hemispheric encoding asymmetry is more apparent than real. J. Cogn. Neurosci. 14, 702–708 10.1162/08989290260138609 12167255

[B35] Nolde S. F. Johnson M. K. Raye C. L. (1998). The role of prefrontal cortex during tests of episodic memory. Trends Cogn. Sci. 2, 399–406 10.1016/S1364-6613(98)01233-9 21227255

[B36] Nowicka A. Tacikowski P. (2011). Transcallosal transfer of information and functional asymmetry of the human brain. Laterality 16, 35–74 10.1080/13576500903154231 19657954

[B37] Nyberg L. Petersson K. M. Nilsson L. G. Sandblom J. Aberg C. Ingvar M. (2001). Reactivation of motor brain areas during explicit memory for actions. Neuroimage 14, 521–528 10.1006/nimg.2001.0801 11467924

[B38] Oldfield R. C. (1971). The assessment and analysis of handedness: the Edinburgh inventory. Neuropsychologia 9, 97–113 10.1016/0028-3932(71)90067-4 5146491

[B39] Parker A. Dagnall N. (2010). Effects of handedness and saccadic bilateral eye movements on components of autobiographical recollection. Brain Cogn. 73, 93–101 10.1016/j.bandc.2010.03.005 20413200

[B40] Prichard E. Propper R. E. Christman S. D. (2013). Degree of handedness, but not direction, is a systematic predictor of cognitive performance. Front. Psychol. 4:9 10.3389/fpsyg.2013.00009 23386836 PMC3560368

[B41] Propper R. E. Christman S. D. (2004). Mixed-versus strong right-handedness is associated with biases towards “remember” versus “know” judgements in recognition memory: role of interhemispheric interaction. Memory 12, 707–714 10.1080/09658210344000503 15724359

[B42] Propper R. E. Christman S. D. Phaneuf K. A. (2005). A mixed-handed advantage in episodic memory: a possible role of interhemispheric interaction. Mem. Cognit. 33, 751–757 16248339 10.3758/bf03195341

[B43] Sontam V. Christman S. D. (2012). Semantic organisation and handedness: mixed-handedness is associated with more diffuse activation of ambiguous word associates. Laterality 17, 38–50 21598173 10.1080/1357650X.2010.529450

[B44] Tulving E. Kapur S. Craik F. I. M. Moscovitch M. Houle S. (1994). Hemispheric encoding/retrieval asymmetry in episodic memory – positron emission tomography findings. Proc. Natl. Acad. Sci. U.S.A. 91, 2016–2020 10.1073/pnas.91.6.2016 8134342 PMC43300

[B45] Welcome S. E. Chiarello C. Towler S. Halderman L. K. Otto R. Leonard C. M. (2009). Behavioral correlates of corpus callosum size: anatomical/behavioral relationships vary across sex/handedness groups. Neuropsychologia 47, 2427–2435 10.1016/j.neuropsychologia.2009.04.008 19383501 PMC2730670

[B46] Witelson S. F. (1985). The brain connection – the corpus callosum is larger in left-handers. Science 229, 665–668 10.1126/science.4023705 4023705

